# *In Vitro* Evaluation of the Antioxidant, 3,5-Dihydroxy-4-ethyl-trans-stilbene (DETS) Isolated from *Bacillus cereus* as a Potent Candidate against Malignant Melanoma

**DOI:** 10.3389/fmicb.2016.00452

**Published:** 2016-04-14

**Authors:** Lekshmi R. Nath, S. N. Kumar, Arya A. Das, Bala Nambisan, A. Shabna, Chellapan Mohandas, Ruby John Anto

**Affiliations:** ^1^Division of Cancer Research, Rajiv Gandhi Centre for BiotechnologyThiruvananthapuram, India; ^2^Agroprocessing and Natural Products Division, Council of Scientific and Industrial Research – National Institute for Interdisciplinary Science and TechnologyThiruvananthapuram, India; ^3^Computational Modeling and Simulation Group, Council of Scientific and Industrial Research – National Institute for Interdisciplinary Science and TechnologyThiruvananthapuram, India; ^4^Division of Crop Protection/Division of Crop Utilization, Central Tuber Crops Research InstituteThiruvananthapuram, India

**Keywords:** 3,5-dihydroxy-4-ethyl-trans-stilbene, apoptosis, antioxidant, skin cancer

## Abstract

3,5-dihydroxy Q1 -4-ethyl-trans-stilbene (DETS) is a natural stilbene, which was first identified as bioactive bacterial secondary metabolite isolated from *Bacillus cereus* associated with a rhabditid entomopathogenic nematode. The present study was intended to investigate the antioxidant and anticancer activity of this compound *in vitro*. Antioxidant activity was investigated by assaying DPPH free radical scavenging, superoxide radical-(O2..) scavenging, hydroxyl radical scavenging and metal chelating activity, which proved that the compound is a powerful antioxidant. The metal chelating activity of DETS was higher than butylated hydroxyanisol (BHA) and gallic acid, two well-known antioxidants. As the molecule exhibited strong antioxidant potential, it was further evaluated for cytotoxic activity toward five cancer cells of various origins. Since the compound has a strong structural similarity with resveratrol (trans- 3,4,5-trihydroxystilbene), a well-studied chemopreventive polyphenolic antioxidant, its anticancer activity was compared with that of resveratrol. Among the five cancer cells studied, the compound showed maximum cytotoxicity toward the human melanoma cell line, [A375, IC_50_: 24.01 μM] followed by cervical [HeLa-46.17 μM], colon [SW480- 47.28 μM], liver [HepG2- 69.56 μM] and breast [MCF-7- 84.31 μM] cancer cells. A375 was much more sensitive to DETS compared to the non-melanoma cell line, A431, in which the IC50 of the compound was more than double (49.60 μM). In the present study, the anticancer activity of DETS against melanoma was confirmed by various apoptosis assays. We also observed that DETS, like resveratrol, down-regulates the expression status of major molecules contributing to melanoma progression, such as BRAF, β-catenin and Brn-2, all of which converge in MITF-M, the master regulator of melanoma signaling. The regulatory role of MITF-M in DETS-induced cytotoxicity in melanoma cells was confirmed by comparing the cytotoxicity of DETS in A375 cells (IC_50_-24.01 μM), with that in SK-MEL-2 (IC_50_-67.6 μM), another melanoma cells which highly over-express MITF-M. The compound arrests the cells at S-G2 transition state of the cell cycle, as resveratrol. Our results indicate that DETS is a powerful antioxidant, having anticancer efficacy comparable with that of resveratrol, and is a potential candidate to be explored by *in vivo* studies and in-depth mechanistic evaluation. To our knowledge, this is the first report on the antioxidant and anticancer properties of DETS.

Microbial secondary metabolites have received considerable attention as they exhibit significant antibiotic and cytotoxic activities (Bérdy, 2012) and are excellent antioxidants (Saurav and Kannabiran, 2012). Oxidative stress resulting from excessive reactive oxygen species (ROS) in the human body is substantially related to the occurrence of various diseases such as cancer, diabetes, inflammation, neurological disorders and cardiovascular diseases (Valko et al., 2007). ROS, especially the hydroxyl (HO^⋅^) and alkoxy (RO^⋅^) radicals, are extremely reactive and cause rapid oxidative damage to many essential biological molecules such as polyunsaturated fatty acids, membrane lipids, and nucleotides in our body, which in turn results in the peroxidation of lipid, and oxidation of carbohydrate and protein (Gulcin, 2012; Sharma et al., 2012; Kim et al., 2014). Moreover, oxidative damages on genetic material initiates the steps involved in mutagenesis, carcinogenesis, and aging (Valko et al., 2007). Several compounds isolated from microbes and plants are rich sources of antioxidants and have been shown to possess excellent anticancer potential (Anto et al., 1995; Abdel-Fattah et al., 2012; Ramasubburayan et al., 2015). Though synthetic antioxidants such as BHA and BHT have been shown to protect the human body from oxidative damage, there has been a great concern regarding their toxicity and carcinogenic side effects (Hwang et al., 2013). Thus, it is very important to identify new sources of safe and inexpensive antioxidants of natural origin. Potent natural antioxidants from microbial sources can replace synthetic antioxidants. Several studies including that of ours have illustrated the antioxidant and anticancer potential of natural stilbenes (Huang et al., 2010; Shukla and Singh, 2011; Kumar et al., 2013; Pangeni et al., 2014).

Cancer continues to be one of the leading causes of human death worldwide, and only modest progress has been made in reducing the morbidity and mortality of this disease (Hail, 2005). Melanoma is a skin cancer that arises from the malignant transformation of melanocytes. Epidemiological studies showed that the incidence of melanoma is increasing at a rate faster than that of any other cancers worldwide (Looi et al., 2013). Melanoma is often characterized by resistance to cytotoxic drugs that contributes to the high morbidity and mortality rates in patients worldwide. This emphasizes the importance of discovering new compounds that are both safe and effective against melanoma. Recently we had reported the antimicrobial activity of 3,5-dihydroxy-4-ethyl-trans-stilbene (DETS), isolated from *Bacillus cereus* associated with rhabditid entomopathogenic nematode (Kumar et al., 2014). In the present study, we have conducted a detailed investigation of the antioxidant potential of this compound and have explored *in vitro*, the relevance of evaluating it as an anticancer agent against malignant melanoma, as our preliminary observations indicate that melanoma cells are highly sensitive to this compound.

## Materials and Methods

### Chemicals and Reagents

1,1-Diphenyl-2-picryl-hydrazyl (DPPH), nicotinamide adenine dinucleotide (NADH), BHA, gallic acid, 2,2′-azino-bis (3-ethylbenzothiazoline-6-sulphonic acid) radical (ABTS) and trichloroacetic acid (TCA), Resveratrol were purchased from Sigma (Sigma–Aldrich GmbH, Sternheim, Germany). Hydrogen peroxide and ethylene diamine tetra-acetic acid (EDTA) were purchased from Sigma–Aldrich (St. Louis, MO, USA). Dulbecco’s modified Eagle’s medium (DMEM) was obtained from Life Technologies (Grand Island, NY, USA); Fetal bovine serum (FBS) from PAN Biotech (GmbH, Aidenbach, Germany); Brn-2 (POU domain, class3, transcription factor 2) and MITF-M (Microphthalmia-associated transcription factor) were purchased from Abcam (Cambridge, UK). β-actin and caspases were purchased from Cell Signaling (Beverly, MA, USA) and antibodies against poly ADP-ribose polymerase (PARP) β-catenin, BRAF(serine/threonine-protein kinase B-Raf) and Annexin V apoptosis detection kit was from Santa Cruz Biotechnology (Santa Cruz, CA, USA). All other reagents were of analytical grade and other chemicals used in this study were of the highest purity.

### Test Compound

**FIGURE 1:**
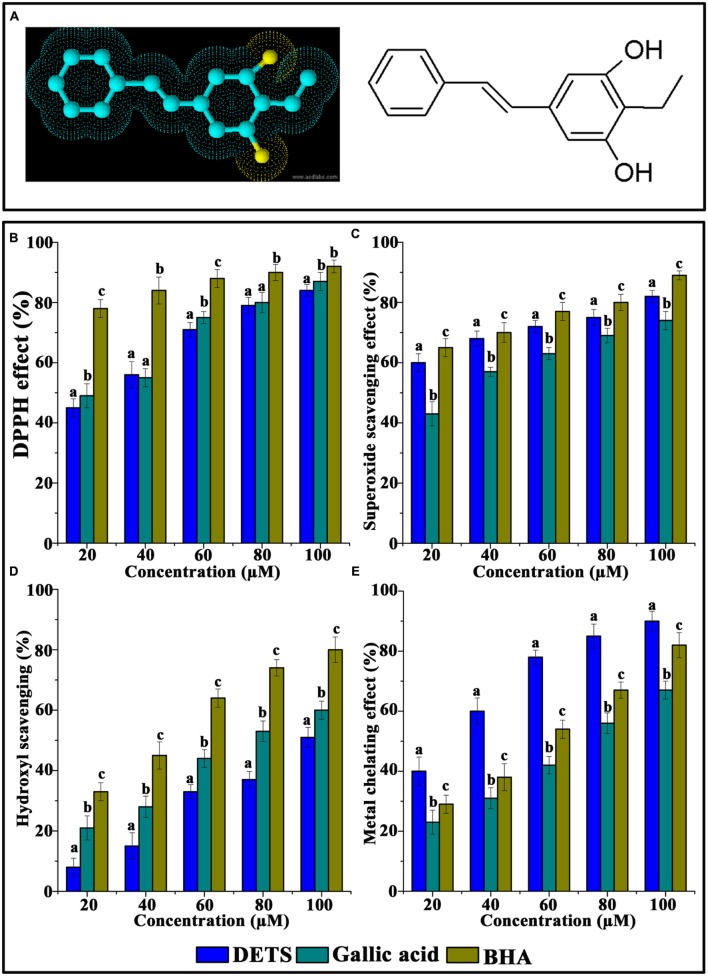
**Chemical structure and antioxidant activity of DETS in various *in vitro* assays.** **(A)** Structure of DETS **(B)** Free radical scavenging, **(C)** superoxide radical-(02-) scavenging assay, **(D)** hydroxyl radical scavenging activity **(E)** metal chelating activity. All the measurements were done in three replicates and results are expressed as arithmetic mean ± standard error on the mean. Different letters in the superscript were significantly different according to Duncan’s multiple range test (*p* < 0.05).

The test compound DETS (**Figure 1A**) was isolated and purified from the cell-free culture filtrate (modified Tryptic soya broth) of a *Bacillus cereus* associated with a rhabditid entomopathogenic nematode and the structure of the compound was established based on detailed spectral analyses (LCMS,^1^H NMR, ^13^C NMR, ^1^H -^1^H COSY, ^1^H -^13^C HMBC) (Kumar et al., 2014). DETS had a strong structural similarity with resveratrol (trans-3,4,5-trihydroxystilbene), a well-known antioxidant and chemopre ventive agent and was selected for a detailed investigation for its antioxidant and anticancer potential.

### Antioxidant Activity

#### DPPH (2′-2′ Diphenyl–2′ Picrylhydrazyl) Radical Scavenging Assay

The free radical scavenging capacity of DETS was measured by the DPPH radical scavenging method of Yen and Chen (1995) with slight modifications. The method involves the reaction of DETS with the stable DPPH in 0.1 mM methanol solution. Briefly, the reaction mixture contained 300 μL of test compound at varying concentrations (20–100 μM) and 2 ml of DPPH solution. After 10 min, the change in absorbance was recorded at 517 nm in a spectrophotometer against a blank, which did not contain the test compound. BHA and gallic acid were used as a positive control. The DPPH radical scavenging capacities were expressed as BHA and gallic acid antioxidant capacity in μg/ml of the test compound.

The % DPPH scavenging activity was calculated by the equation:

$$DPPH\,\;Scavenging\,\;Effect(\%)=[\frac{\left(A0-A1\right)}{A0}\times 100],$$

Where A0 is the absorbance of the control reaction and A1 is the absorbance in the presence of the test compound or standards. In order to calculate the IC_50_ value, which is the amount of sample necessary to decrease the absorbance of DPPH radical by 50%, the decolourization was plotted against the concentration of stilbene.

#### Superoxide Radical-(O2-)-Scavenging Assay

The assay was based on the capacity of the antioxidant to inhibit formazan formation by scavenging the superoxide radicals generated in the riboflavin-light-NBT system (Fridowich, 1995). The method used by Martinez et al. (2001) for determination of superoxide dismutase was followed by modifications. Each 3 ml of reaction mixture contained 50 mM sodium phosphate buffer, pH 8.0, 13 mM methionine, 2 μM riboflavin, 100 μM EDTA, NBT (75 μM), and 1 ml of the DETS of different concentrations. The production of blue formazan was followed by monitoring the increase in absorbance at 560 nm after a 10 min illumination from a fluorescent lamp. The entire reaction assembly was enclosed in a box lined with aluminum foil. Identical tubes with reaction mixture were kept in the dark and served as blanks. The percentage inhibition of superoxide anion generation was calculated using the following formula:

$$(\%)Inhibition=\frac{Ac-As}{Ac}\times 100$$

Where Ac is the absorbance of the control and As is the absorbance of the samples.

### Hydroxyl Radical Scavenging Activity

The hydroxyl radical-scavenging activity was conducted using the 2-deoxyribose method (Halliwell et al., 1987). Briefly, the assay mixture contained 2.8 mM 2-deoxyribose, 20 μM ferrous ion solution, 100 μM EDTA, and different sample concentrations (10–100 μM) in a total volume of 1 ml of 10 mM potassium phosphate buffer (pH 7.4). All the components were dissolved in 10 mM phosphate buffer (pH 7.4). The ferrous iron solution and EDTA were premixed before they were added to the assay mixture. The reaction was started by the addition of a mixture of 1.42 μM H_2_O_2_ and 100 μM ascorbate. The mixture was incubated at 37°C for 30 min. At the end of the incubation time, 1 ml of 1% (*w*/*v*) TBA in 50 mM sodium hydroxide and 1 mL of 2.8% (*w*/*v*) TCA were added and the mixture was heated for 30 min in a boiling water bath, cooled, and the absorbance at 532 nm was measured, which corresponds to deoxyribose damage. BHT and gallic acid were used as a positive control. All experiments were conducted in triplicate.

The inhibition percentage (I%) of the radical-scavenging capacity was calculated using the following equation:

$$1\%=\frac{\left[\left(Ahydroxyl-Ablank\right)-\left(As-hydroxyl-As-blank\right)\right]}{\left(Ahydroxyl-Ablank\right)}\times 100$$

Where *A*hydroxyl is the absorbance of the hydroxyl solution, *A*blank is the absorbance of methanol instead of the hydroxyl solution, *A*s-hydroxyl is the absorbance of the hydroxyl solution in the presence of sample, and *A*s-blank is the absorbance of methanol in the presence of the sample. IC_50_ values, which represent the concentration of the sample that caused 50% hydroxyl radical-scavenging activity, were calculated from the plot of inhibition percentage against sample concentration.

### Metal Chelating Activity

The chelation of ferrous ions by the DETS was estimated by the method of Dinis et al. (1994) and Decker (1997) with slight modifications and compared with that of BHA and gallic acid. The chelation test initially includes the addition of ferrous chloride. The antioxidant present in the sample chelates the ferrous ions from the ferrous chloride. The remaining ferrous combines with ferrozine to form ferrous–ferrozine complex. The intensity of the ferrous–ferrozine complex formation depends on the chelating capacity of the sample and the color formation was measured at 562 nm (Shimadzu UV-Vis 2450, Shimadzu Corporation).

Different concentrations of DETS and standard (20–100 μM) were added to a solution of 100 ml FeCl_2_ (1 mM). The reaction was initiated by the addition of 200 ml ferrozine (1 mM). The mixture was finally quantified to 1.3 ml with methanol, shaken vigorously and left standing at room temperature for 10 min. After the mixture had reached equilibrium, the absorbance of the solution was measured spectrophotometrically (Shimadzu UV-Vis 2450, Shimadzu Corporation). The percentage inhibition of ferrous–ferrozine complex formation was calculated using the formula.

$$Percentage\,\;of\,\;chelation=\frac{Ac-As}{Ac}\times 100$$

Where “Ac” is the absorbance of control, “As” is the absorbance of the sample. Percentages of RSA were plotted against the corresponding concentration of the extract to obtain IC_50_ value and were expressed in terms of mg/ml.

### Anticancer Activity

#### Cell Culture and Cell Viability

The cancer cells of different origins were used in the study. (I) Breast cancer cell line (MCF-7), (II) cervical cancer cell line (HeLa), (III) liver cancer cell line (HepG2) (IV) colon cancer cell line (SW480) and (V) skin cancer cell lines (A375), (A 431), (SK-MEL-2) were purchased from National Centre for Cell Science, Pune, India and maintained in DMEM supplemented with 10% FBS with antibiotics and antimycotics at 37°C in a CO_2_ incubator at Division of Cancer Research Program, Rajiv Gandhi Centre for Biotechnology (RGCB), Thiruvananthapuram. All the experiments on cancer cell lines were carried out in the above laboratory.

#### MTT Assay

The cytotoxic activity of the compound was assessed by standard MTT assay as described earlier (Smitha et al., 2005). Briefly, the cells were seeded in 96-well plates (2000 cells/well), incubated overnight, treated with DETS (10–250 μM) for 48 h in five different cancer cells. The most sensitive cell line A375, was treated with different concentrations of the compound (5–50 μM) for 24, 48, and 72 h, keeping untreated controls. The cytotoxicity of this compound was compared with that of resveratrol (5–50 μM). For comparison between A375 and A431 and between A375 and SK-MEL-2, cells were treated with different concentrations of DETS (10–50 μM) and (10–100 μM) respectively. For this fresh media containing 25 μl of MTT solution (5 mg/ml in PBS) and 75 μl of complete medium was added to the wells and incubated for 2 h. At the end of incubation, MTT lysis buffer (20% sodium dodecyl sulfate in 50% dimethylformamide) was added to the wells (0.1 ml/well) and incubated for another 1 h at 37°C. At the end of incubation, the optical density was measured at 570 nm using a plate reader (Bio-Rad).

The relative cell viability in percentage was calculated as

$$\left(\frac{Absorbance\,\;of\,\;test\,\;samples}{Absorbance\,\;of\,\;control\,\;samples}\right)\times 100.$$

The IC_50_ values were extrapolated from polynomial regression analysis of experimental data.

#### Phase-Contrast Microscopy

A375 cells were plated at a density 10,000 cells/well into a 24 well plate and treated with 25 μM DETS for 72 h. Cells were viewed by phase-contrast light microscope (Nikon, TMS, Japan) and photographs were taken using a Nikon camera (Japan) (Kumar et al., 2013).

#### Acridine Orange/Ethidium Bromide Staining

Morphological changes characteristic of apoptosis were assessed by fluorescent microscopy using acridine orange/ethidium bromide staining method. Briefly, cells were seeded in 96-well plates and treated with DETS as in MTT assay, but for 24 h. After washing once with PBS, the cells were stained with 100 μl of a 1:1 mixture of acridine orange–ethidium bromide (4 μg/ml) solutions, immediately washed with PBS and photomicrographed under a Nikon inverted fluorescent microscope (TE-Eclipse 300) (Oommen et al., 2004).

#### Detection of Apoptosis by Annexin V–PI Staining by Fluorescence Microscopy

As apoptosis causes changes in membrane permeability, there is a transient leakage of phosphatidylserine to the membrane, which is considered to be an early marker of apoptosis. Annexin preferentially binds to phosphatidylserine as it is a negatively charged phospholipid. Hence using FITC conjugated annexin V, apoptotic cells were detected with the help of a fluorescent microscope by manufacturer’s protocol (Santa Cruz, CA, USA). Briefly, the cells were seeded in 96-well plates and treated with the DETS as in MTT assay, but for 24 h. The cells were first washed with PBS and then with 1 × assay buffer after which, 0.5–5 μl (0.1–1 μg) of Annexin V FITC per 100 μl assay buffer was added. After incubating for 15 min at room temperature in the dark, the cells were washed with PBS and immediately photographed using a fluorescence microscope (Laladhas et al., 2010).

#### Western Blot Analysis

For the detection of apoptotic proteins, A375 cells (0.7 × 10^6^ cells/60 mm culture dish) were treated with DETS (15 and 25 μM) and resveratrol (20 μM) for 24 h after which, the cells were washed with PBS and lysed by keeping on ice for 30 min with whole cell lysis buffer containing 20 mM Tris (pH 7.4), 250 mM NaCl, 2 mM EDTA, 0.1% Triton, 1 mM DTT, 0.5 mM PMSF, 4 mM sodium orthovandate, aprotinin (5 μg/ml) and leupeptin (5 μg/ml). The supernatants were collected by centrifuging at 13,000 *g* for 10 min at 4°C and boiling in 5× loading dye before separating the proteins by SDS-polyacrylamide gel electrophoresis (SDS-PAGE) and Western blotting them using antibodies against caspases (caspase 3, caspase 7, caspase 8, and caspase 9), poly (ADP-ribose) polymerase (PARP), melanoma specific molecules such as BRAF, MITF-M, β-catenin and Brn-2. Immunoreactive proteins were detected with horseradish peroxidase coupled secondary antibodies and visualized by enhanced chemiluminescence detection kit (Millipore Corporation, Billerica, MA, USA) (Anto et al., 2003).

#### Flow Cytometry and Cell Cycle Analysis

Cell cycle analysis helps in distinguishing the distribution of a population of cells in the various stages of cell cycle. Briefly, cells were treated with DETS as well as resveratrol, which served as the positive control for 48 h followed by trypsinization. The cell pellets were fixed in 70% ice–cold ethanol, treated with 100 mg/ml RNAase A and 50 mg/ml propidium iodide, followed by flow cytometric analysis (BD Biosciences) (Sreekanth et al., 2011).

### Molecular Docking

Molecular Docking experiment of DETS into the β-catenin ligand binding domain was done using the software’s Autodock 4.2 and iGEMDOCK v2.1 (Mahindroo et al., 2006; Morris et al., 2009; Hsu et al., 2011). These docking software’s were used to find the appropriate binding and conformations of the ligand to the receptor. The 3D model of β-catenin (PDB id: 4DJS) was retrieved from the Brookhaven Protein Data Bank (PDB)^1^. DETS (ChemSpider ID: 4943923), the structure was downloaded from PubChem^2^ and converted to DETS PDB file using Chem3D Pro 10.

### Statistical Analyses

Statistical analyses were performed with the SPSS software package (Version 17.0; SPSS, Inc., Chicago, IL, USA). Statistical significance was defined as *p* < 0.05. All values are expressed as mean ±SD of three parallel measurements.

## Results

### Antioxidant Activity

The DPPH radical-scavenging activity was investigated at different concentrations (20–100 μM) of the DETS. The results presented in **Figure 1B** clearly demonstrated that the DETS exhibited an interesting radicals scavenging activity with an IC_50_ value of 40 μM.

**Figure 1C** shows the superoxide radical-(O2-)-scavenging activity of the DETS, as measured by the riboflavin-NBT light system *in vitro*. The DETS was found to be a potent scavenger of superoxide radical generated in riboflavin-NBT-light system *in vitro*. The DETS inhibited the formation of the blue formazan and the % of inhibition was proportional to the concentration with an IC_50_ value of 20 μg/ml and had a notable effect on scavenging of superoxide when compared with BHA and gallic acid, which was used as a positive control. These results indicated that the tested DETS recorded significant the superoxide radical scavenging activity and this activity are all most comparable with that of BHA (**Figure 1C**).

**Figure 1D** shows the hydroxyl radical scavenging effects of DETS determined by the 2-deoxyribose oxidation method. DETS recorded very significant scavenging properties against hydroxyl radicals, and the inhibition percentage was proportional to the concentration of the compound. At 20–100 μM concentrations of DETS, hydroxyl radical scavenging activity of the DETS was higher than BHA and gallic acid. The chelating property of DETS was studied against Fe^2+^. The chelating ability of the DETS is shown in **Figure 1E** and chelating activity was also better than BHA and gallic acid (**Figure 1E**).

### Anticancer Activity

#### DETS Induces Maximum Cytotoxicity in Skin Cancer Cells

**FIGURE 2:**
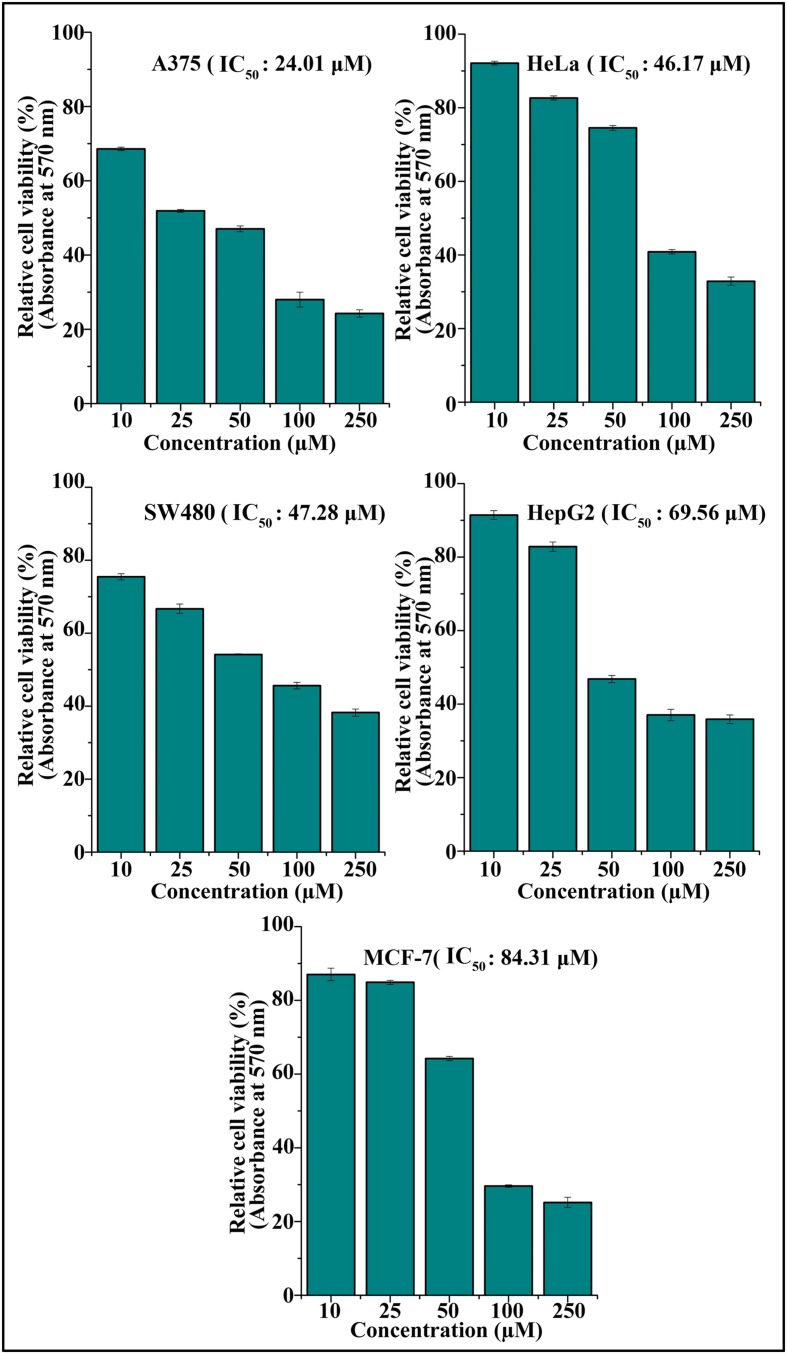
**DETS induces maximum cytotoxicity in skin cancer cells.** Effect of DETS on cancer cells of various origin. A total of 2000 cells in triplicate were exposed to the indicated concentration of DETS (10–250 μM) for 48 h and subjected to MTT assay. Relative cell viability was determined as % absorbances over untreated control. All the measurements were done in three replicates and results are expressed as arithmetic mean ± standard error on the mean. Different letters in the superscript were significantly different according to Duncan’s multiple range test (*p* < 0.05).

**FIGURE 3:**
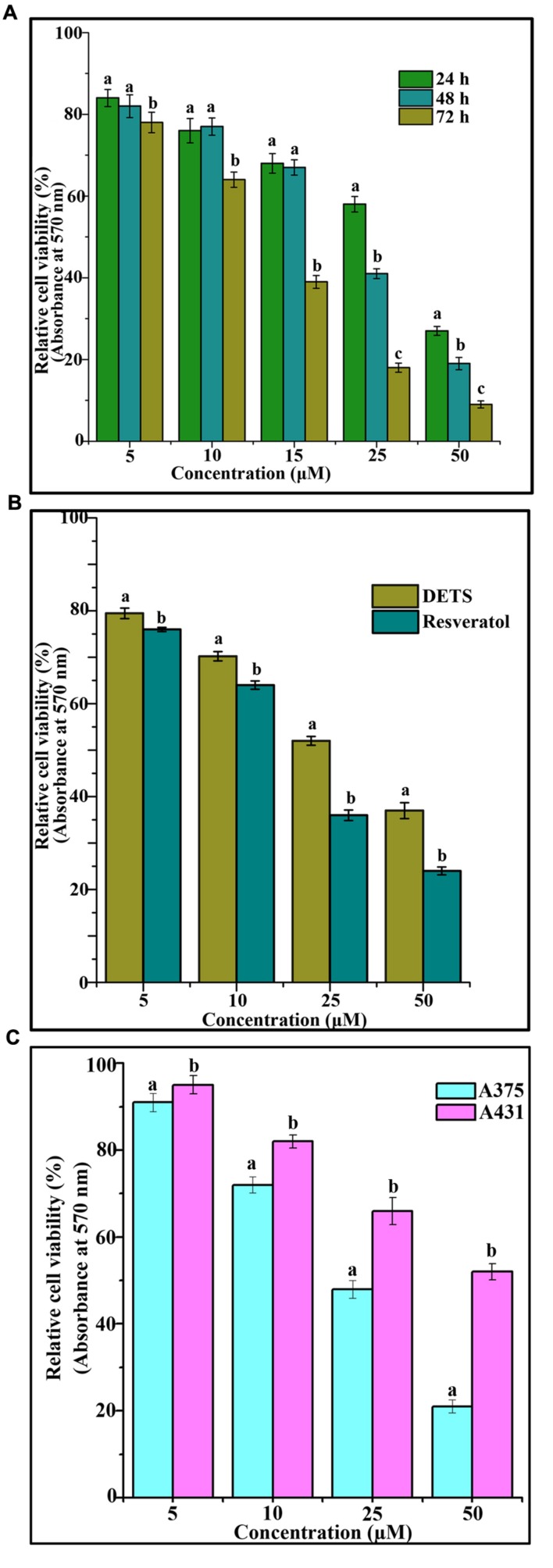
**DETS induces maximum cytotoxicity in melanoma cells.** **(A)** Time dependent effect of DETS against human skin cancer cell, A375. **(B)** Comparison with resveratrol. All the measurements were done in three replicates and results are expressed as arithmetic mean ± standard error on the mean. **(C)** Comparison of the cytotoxicity of DETS in A375 (melanoma cell line) with that of a A431 (non-melanoma cell line). Cells were seeded in 96 wells and treated with indicated concentrations of DETS and resveratrol and subjected to MTT assay. All the measurements were done in three replicates and results are expressed as arithmetic mean ± standard error on the mean. Different letters in the superscript were significantly different according to Duncan’s multiple range test (*p* < 0.05).

The cytotoxic effect of DETS was evaluated in five cancer cells of various origin (colon, breast, skin, liver, and cervical) (**Figure 2**), among which the compound was found to be most active in the skin cancer cell, A375 (IC_50_: 24.01 μM) (**Figure 2**) followed by HeLa (IC_50_: 46.17 μM), SW480 (IC_50_: 47.28 μM), HepG2 (IC_50_: 69.56 μM) and MCF-7 (IC_50_: 84.31 μM). The most sensitive A375 cell was treated with different concentrations of the compound for 24 h, 48 and 72 h as indicated in **Figure 3A** and the cell viability was determined by MTT assay. We observed a dose- dependent and time-dependent induction of cytotoxicity by DETS, which was comparable with that of resveratrol having structural similarity. While resveratrol is having an IC_50_ of 20.19 μM, DETS showed an IC_50_ of 24.01 μM after 48 h of drug treatment (**Figure 3B**). We also compared the cytotoxicity of DETS in A375 with that of a non-melanoma cell line, A431. It was interesting to see that the IC_50_ of the compound was more than double in this cell line compared to the melanoma cells (49.60 μM) (**Figure 3C**).

#### DETS Induces Morphological Changes, Nuclear Membrane Damage and Membrane Flip-Flop Characteristic of Apoptosis in A375

**FIGURE 4:**
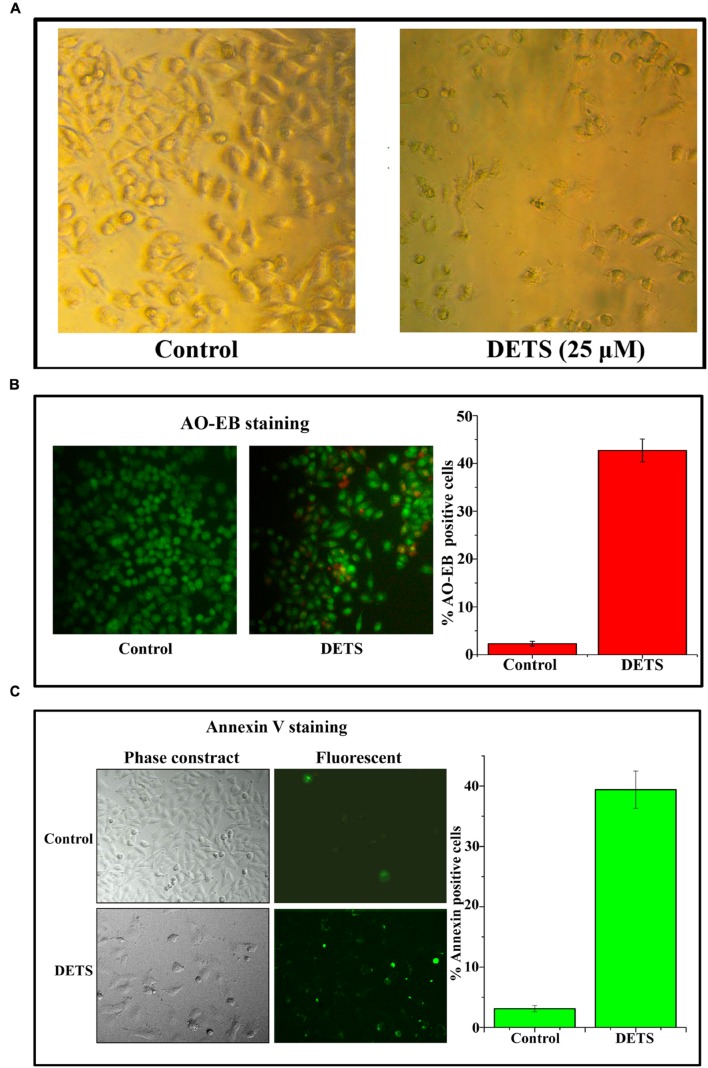
**DETS induces morphological changes, membrane damage and membrane flip-flop, characteristics of apoptosis in melanoma cell line, A375.** **(A)** Phase-contrast light microscopy image of A375 cells after 72 h treatment. **(B)** The early stage and late stage of apoptosis was evaluated by Acridine orange/ethidium bromide staining. AO/EB positive cells were counted in different fields and the average was taken and plotted. **(C)** A375 cells were treated as indicated with DETS for 24 h and stained for annexin-V positivity and membrane flip-flop was captured by fluorescence microscopy. Annexin-V positive cells in various fields were counted, and the average was taken and plotted. Cells were seeded in 96 well plate, treated with DETS for 24 h and subjected to apoptosis assays. Error bars indicate the standard deviations of 3 measurements. Different letters in the superscript were significantly different according to Duncan’s multiple range test (*p* < 0.05).

The cultured A375 cells were examined for their morphology after 72 h treatment with DETS. It was observed that the compound is inducing typical morphological changes such as nuclear condensation, membrane blebbing and formation of apoptotic bodies, characteristic of apoptosis, compared to untreated control as assessed by phase contrast microscope. Moreover, there was a significant reduction in the number of cells after drug treatment (**Figure 4A**). AO/EB staining was done to confirm the nuclear membrane damage, a characteristic feature of apoptosis as observed by yellow/orange coloration in the nuclei of cells treated with DETS. The cells treated with the compound after 24 h exhibited 45% AO-EB positivity, whereas only 2.3% cells were AO-EB positive in the untreated control (**Figure 4B**). Bright green annexin fluorescence was imparted to membranes of the apoptotic cells revealing membrane flip-flop an indicator of the early stage of apoptosis. The cells treated with DETS produced 39% annexin V positivity, while the untreated cells produced only 3.01% positivity (**Figure 4C**).

#### DETS Induces Caspase-Dependent Apoptosis in A375 Cells Leading to PARP Cleavage

**FIGURE 5:**
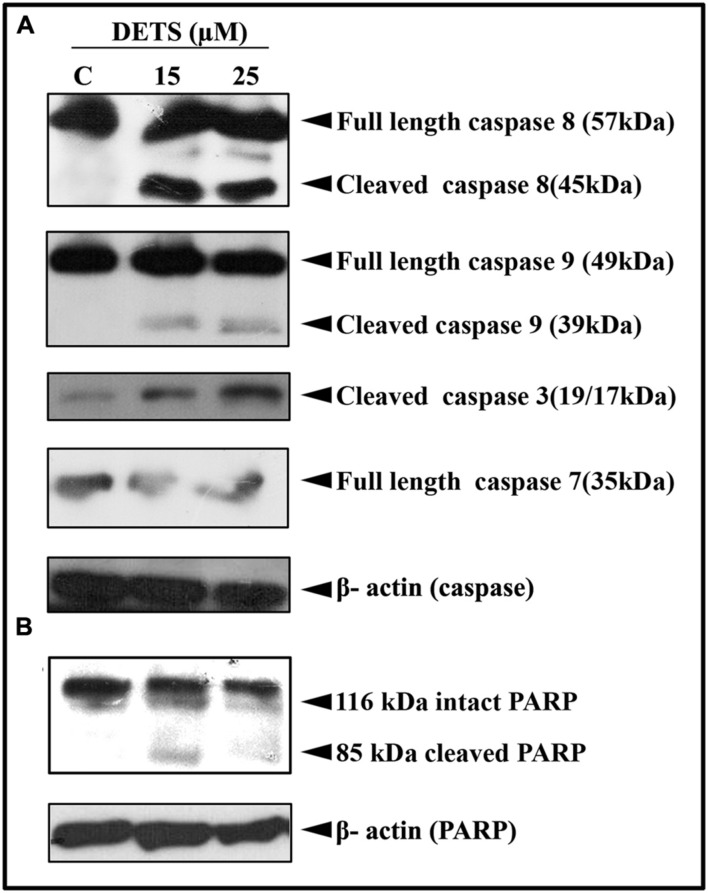
**DETS induces caspase-dependent apoptosis.** **(A)** In A375 cells leading to PARP cleavage. **(B)** Whole-cell extracts were prepared after treating A375 cells with indicated concentration of DETS for 24 h and subjected to Western blotting using antibodies against the caspases, 8, 9, 7, and 3 and PARP. All results shown here are representative of three independent experiments with similar results.

Our next attempt was to investigate the mechanism behind the cytotoxic effect of DETS. First, we checked the role of caspases, the key regulators of the apoptotic program. We observed that DETS induces dose-dependent cleavage of the initiator caspases, caspase 8 and 9 (**Figure 5A**), which clearly indicates the role of mitochondria in DETS-induced apoptotic program in A375 cells. The compound also brought about a significant cleavage of the effector caspases, caspase 3 and 7 (**Figure 5A**). Then we checked the effect of DETS on the DNA repair enzyme PARP (Poly ADP-Ribose polymerase (116 kDa), the down-stream target of caspases 3 and 7, which cleave it into fragments of 85 kDa. As observed in **Figure 5B**, while 116 kDa PARP remained intact in the untreated cells, DETS cleaved the intact PARP to its fragments, clearly indicating caspase-mediated apoptosis.

#### DETS Induce S-G2M Transition Cell Cycle Arrest in A375 Cells

**FIGURE 6:**
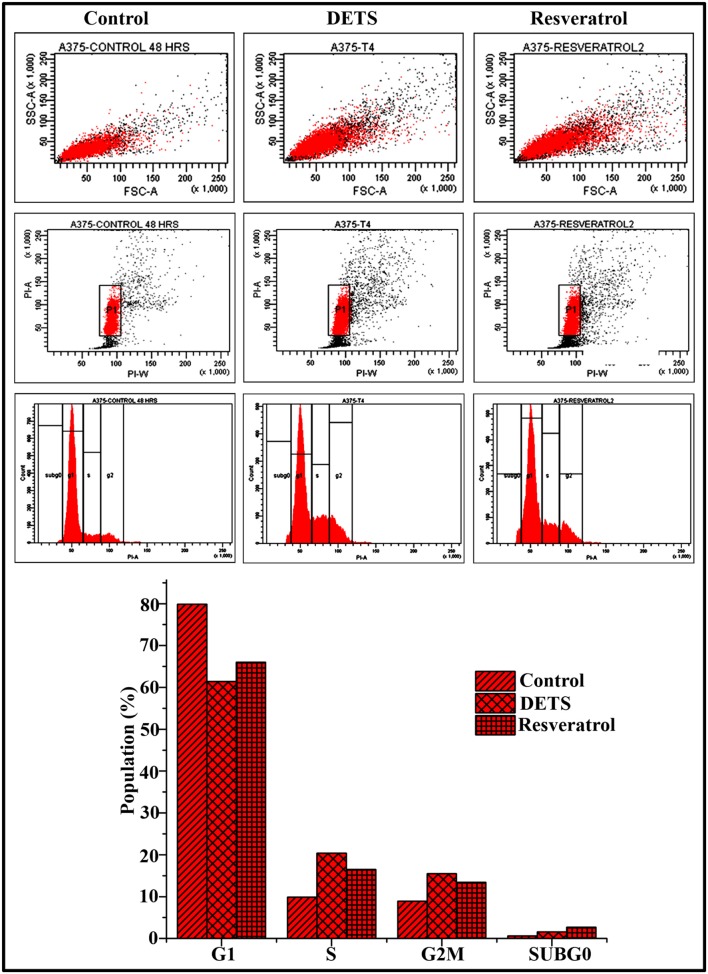
**DETS induce S-G2 M cell cycle arrest in A375 cells.** Cells were harvested after 48 h of sample treatment, fixed in alcohol, stained with propidium iodide, and assayed for DNA content by flow cytometry. Representative histograms indicate the percentages of cells in G1, S, G2/M and sub G0 phases of the cell cycle. The percentage of cells with sub-G0 DNA content was taken as a measure of the apoptotic cell population. Resveratrol (20 μM) was used as positive control. The data provided is representative of three independent experiments. Bars with different characters are statistically different at *p*, 0.05 level.

To explore whether the growth-inhibitory effect of DETS on A375 cells is mediated through cell cycle arrest, we analyzed the distribution of cells in different phases of the cell cycle, by measuring intracellular DNA content in each phase. It was very interesting to note that, DETS induced a significant S-G2 transition arrest in A375 cells, as effectively as the positive control, resveratrol (20 μM) after 48 h of treatment. Treatment with 25 μM of DETS increased population in S and G2/M phase with an accumulation of 20.4 and 15.5% respectively while 20 μM resveratrol induced an accumulation of 16.5 and13.4% respectively compared to 9.9 and 8.9% in the untreated cells (**Figure 6**).

#### DETS Down-Regulates Survival Signals Prevalent in Melanoma

**FIGURE 7:**
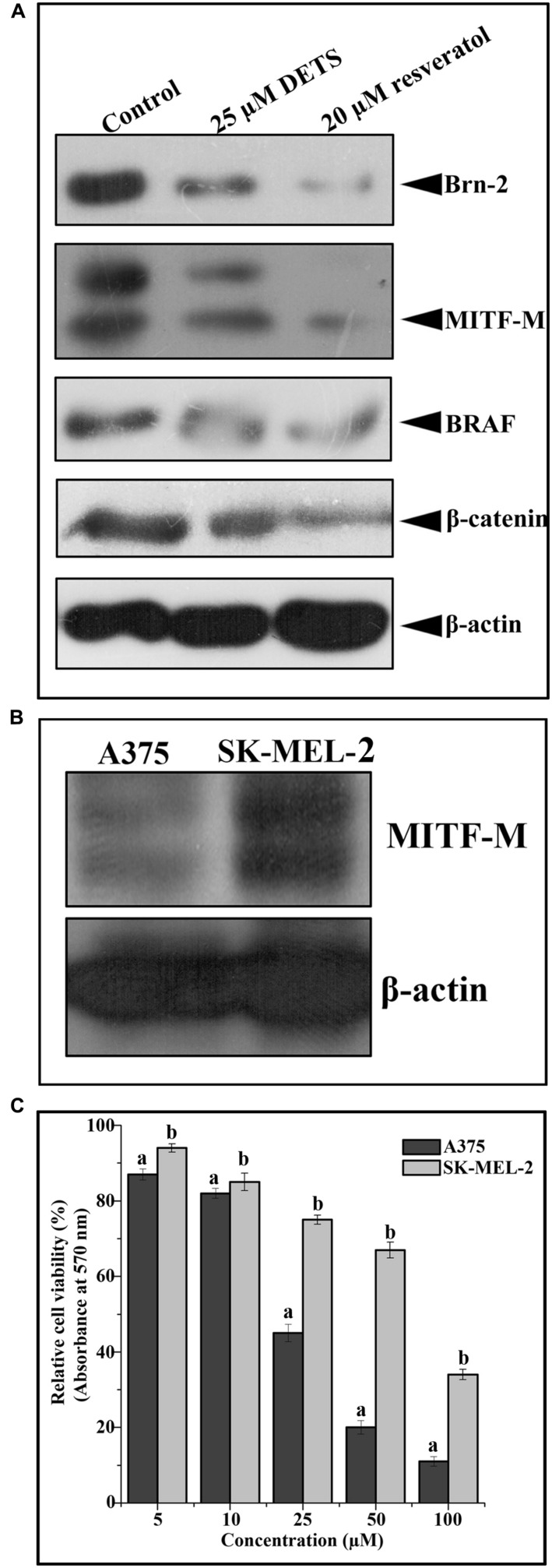
**DETS down-regulates survival signals prevalent in melanoma.** **(A)** DETS down-regulates activation of survival signals such as BRAF, Brn-2, β -catenin and MITF-M. Whole-cell extracts were prepared after treating A375 cells with indicated concentration of DETS as well as resveratrol for 24 h and subjected to Western blotting using antibodies against BRAF, Brn-2, β -catenin and MITF-M. **(B)** The expression status of MITF-M in A375 and SK-MEL-2. Whole-cell extracts were prepared with A375 and SK-MEL-2 cells and subjected to Western blotting using antibodies against MITF-M and β-actin. **(C)** Comparison of two melanoma cells A375 with SK-MEL-2. Cells were seeded in 96 well plate, treated with DETS for 48 h and subjected to MTT assay. All results shown here are representative of three independent experiments with similar results.

We analyzed the effect of DETS on the key regulatory molecules of melanoma signaling and compared it with that of resveratrol, a structurally similar anticancer compound. We observed that DETS is effectively down-regulating the expression of oncogenic BRAF which is shown to be mutated and activated in A375 cells (**Figure 7A**) and Brn-2, a molecule highly over-expressed in BRAF mutant melanoma cells and (**Figure 7A**). β-catenin, the down-stream molecule of Wnt signaling was also significantly abrogated upon 24 h of treatment with DETS (**Figure 7A**). It was very interesting to see that, DETS also down-regulated the constitutively expressed MITF-M, the master regulator of melanoma progression, though not as effectively as resveratrol (**Figure 7A**). To confirm the regulatory role of MITF-M in the anticancer potential of DETS, we compared the cytotoxicity of the molecule in A375 with that of SK-MEL-2, another melanoma cell line, which highly over-express MITF-M (**Figure 7B**. Illustrating our hypothesis, these cells were highly resistant to DETS (IC_50_-67.6 μM) (**Figure 7C**).

**FIGURE 8:**
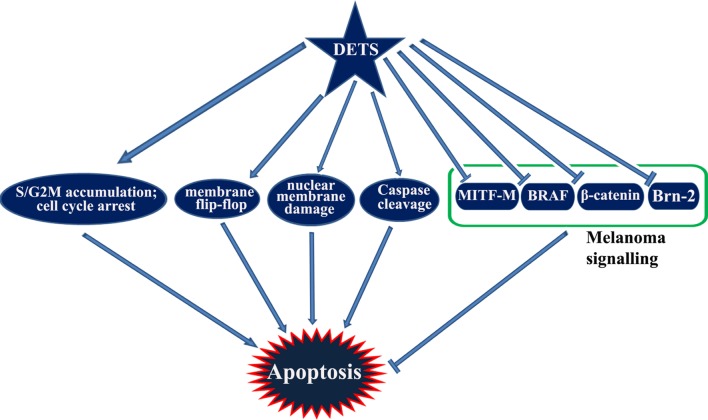
**A graphical representation of the study**.

Taken together, DETS, a potent antioxidant isolated from the cell-free culture filtrate of a *Bacillus cereus* associated with a rhabditid entomopathogenic nematode induces cell cycle arrest as well as apoptosis in melanoma cells and inhibits the constitutive expression of melanoma specific molecules. It is not clear from this study whether DETS is inhibiting all these molecule independently. As A375 is a BRAF activated cell line, inhibition of BRAF by DETS may be leading to the inhibition of the down-stream molecules Brn-2 and β-catenin that leads to the inhibition of MITF-M, the pivotal molecule regulating the proliferation of melanoma. Further studies are going on to identify the mechanism of action of this molecule. **Figure 8** illustrates the results of the present study.

#### Docking of DETS to β-Catenin

**FIGURE 9:**
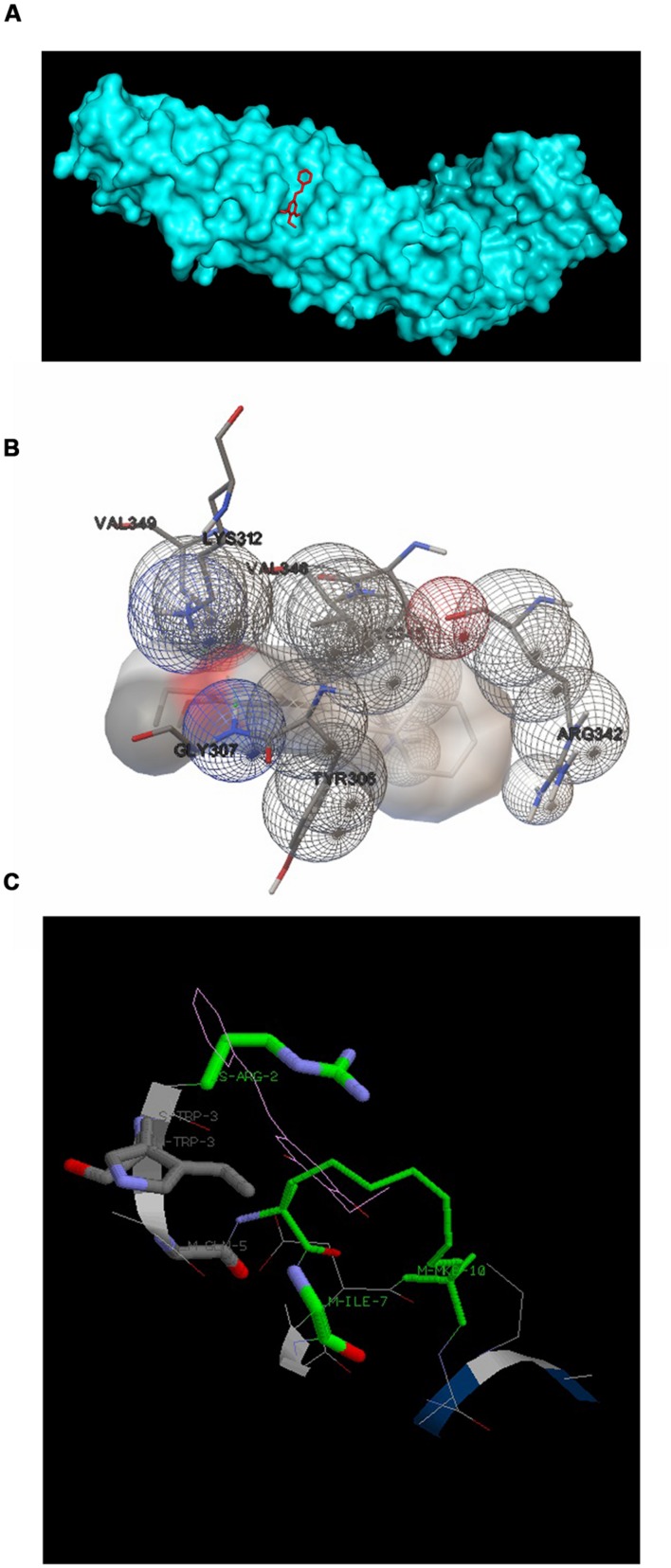
**Docking configurations of DETS in to β-catenin.** Docking simulation was performed to identify interaction between DETS in to β-catenin using the Software: Autodock 4.2 and iGEMDOCKv2.1 **(A)** and **(B)** Docking modes of DETS on β-catenin using Autodock 4.2. **(C)** The best docking pose of DETS to β-catenin using iGEMDOCK.

3,5-dihydroxy-4-ethyl-trans-stilbene was made to bind to β-catenin (PDB id:4DJS) and the free energy of binding was determined as -5.82 kcal/mol, showing the high potential binding affinity into the binding site (**Figures 9A,B**). The visualization was also done using PYMOL (The PyMOL Molecular Graphics System, Version 1.7.4 Schrödinger, LLC). The docking fitness of the ligand molecules to β-catenin and the amino acids of the receptor (β-catenin) involved in interaction was predicted by the iGEMDOCK using default docking parameters (**Figure 9C**). The interaction table and the binding energy of β-catenin were analyzed using the iGEMDOCK and was shown in **Table 1**.

**Table 1:** Table represented the fitness and Interaction values during the molecular docking of 3 DETS in to β-catenin.

(A)				

**Compound**	** Energy**	** VDW**	** HBond**	**Elec**
P-catenin-4943 923-0.pdb	-64.0896	-46.7634	-17.3262	0

**(B)**				

**Compound**		**P-catenin-4943923**		
			
Energy		-64.1
H-S-ARG-2		-6.41676
H-M-ILE-7		-3.5
H-M-MK8-10		-7.40941
V-S-ARG-2		-8.68679
V-M-TRP-3		-4.09979
V-S-TRP-3		-12.6087
V-M-GLN-5		-5.72614
V-M-MK8-10		-4.39502


***(A)** Fitness table and **(B)** Interaction table.*

## Discussion

3,5-Dihydroxy-4-ethyl-trans-stilbene (also named 2-isopropyl-5-(2- phenylethenyl)-benzene-1,3-diol) belonged to the stilbene family and was first identified as a bacterial metabolite with significant antimicrobial activity from entomopathogenic nematode (Richardson et al., 1988; Hu et al., 1998). Recently we also have reported this compound from a *Bacillus cereus* associated with rhabditid entomopathogenic nematode (Kumar et al., 2014). Interestingly, this compound has a structural similarity to 3,5-Dihydroxy-4-isopropylstilbene, a stilbene previously reported from *Photorhabdus* spp. and *Xenorhabdus* spp. associated with entomopathogenic nematodes and has attracted much attention to many researchers worldwide for its diversified biological and pharmacological properties, including anti-inflammatory and immunomodulatory (Hu and Webster, 2000; Tang et al., 2007). In the present study, we have noted that DETS also possess significant antioxidant and anticancer property.

More than half of the currently available drugs are natural or related compounds (Hagiwara et al., 2012). Overproduction of free radicals may increase in our body due to pollution and other external factors, and their removal by our natural antioxidant systems may be lower than before due to a number of factors related to our lifestyle. However, the production of free radicals can be balanced by antioxidant activity of endogenous enzymes as well as natural and synthetic antioxidants (Volka et al., 2006). Antioxidants exert its action through several mechanisms including prevention of chain initiation, chelating of transition metal ion catalysts, decomposition of peroxidases, prevention of continued hydrogen abstraction and radical scavenging (Islam et al., 2013).

The effect of antioxidants on DPPH radicals is thought to be due to their hydrogen donating ability (Islam et al., 2013). In the present study DETS, the free OH group may act as hydrogen donor and this may be one of the reasons for the antioxidant property of this compound. Radical scavenging activities are very important to prevent the harmful role of free radical in various diseases including cancer. DPPH free radical scavenging is a well- accepted mechanism by which antioxidants act to inhibit lipid peroxidation. DPPH method has been used very much extensively to predict antioxidant activities of various compounds and extracts because of the relatively short time required for analysis. The mutagenic property of free radicals is due to the direct interaction of hydroxyl radicals with DNA and therefore plays an important role in carcinogenesis (Baumann et al., 1979). Hence, this indicates that the phenolic content is positively correlated with DPPH radical scavenging activity and superoxide anion scavenging activity. As phenolic compounds have redox properties, this result is hardly surprising. The radical scavenging activity is usually related to the presence of hydroxyl substituents in aromatic rings, which contribute to their hydrogen donating activity (Phang et al., 2013). Attesting this information, we also noted significant DPPH activity by DETS.

Hydroxyl radicals can be generated by the biochemical reaction. Superoxide radical is eventually converted by superoxide dismutase to hydrogen peroxide, which can subsequently produce extremely reactive hydroxyl radicals. DETS recorded significant hydroxyl radical scavenging property. The evaluation of the metal chelating activity becomes important as it reduces the concentration of the catalyzing transition metal in lipid peroxidation (Jayamurthy et al., 2013). Studies have reported that chelating agents, which form bonds with a metal, are effective as secondary antioxidants because they reduce the redox potential, thereby stabilizing the oxidized form of the metal ion (Jayamurthy et al., 2013). Phenolic compounds have been recognized to possess high antioxidant properties. The antioxidant activity of phenolic compounds is mainly due to their redox properties, which allow them to act as radical scavengers, metal chelators, reducing agents, hydrogen donors and singlet oxygen quenchers (Phang et al., 2013). The present compound, DETS possess a free OH group, which may be one of the reasons for its significant antioxidant property.

Previously 3,4,5-trihydroxystilbene (Resveratrol), a compound that is similar to DETS has been reported for antioxidant activity and is both a free radical scavenger and a potent antioxidant because of its ability to promote the activities of a variety of antioxidant enzymes. The ability of the polyphenolic compounds to act as antioxidants depends on the redox properties of their phenolic hydroxyl groups and the potential for electron delocalization across the chemical structure (Subramanian et al., 2015). To the best of our knowledge, this is the first report on the antioxidant activity of DETS.

Several natural products having antioxidant potential have been shown to be potent anticancer agents (Vinod et al., 2013; Patra et al., 2015). The present study also implies that DETS, which is structurally similar to the well known antioxidant and anticancer compound, resveratrol, is a very good antioxidant as well as the anticancer agent. Natural products seem to have gained attention worldwide for the management of neoplasia and certain precancerous conditions.

Skin cancer is the most common cancer in the United States, where one in five people are affected with skin cancer during their lifetime (Robinson, 2005; Stern, 2010) and the average annual cost for treating skin cancer comes around $8.1 billion (Guy et al., 2015a). Melanoma is the deadliest form of skin cancer, which arises due to the malignant transformation of melanocytes. It has been reported that melanoma incidence in the United States has doubled from 1982 to 2011 (Guy et al., 2015b) and estimates indicate that, by 2015, one in 50 Americans will develop melanoma in their lifetime (Rigel et al., 2010). Earlier reports by The World Health Organization also claim that every year more than 65,000 people die of melanoma, worldwide (World Health Organization [WHO], 2006). Our study indicates that DETS induces maximum cytotoxicity in melanoma cells among cancer cells of different origins. The current treatment modalities have been proven to be inadequate for the management of this cancer. Therefore, there is an urgent need to develop mechanism-based novel approaches for prevention/therapy of skin cancer. The present study documents that DETS, which belongs to the stilbene family and hence, having structural similarity to resveratrol, induces apoptotic mode of cell death in melanoma cells. Resveratrol has been shown to induce cytotoxicity and apoptosis in different types of malignant cell lines, including melanoma (Clement et al., 1998; Fuggeta, 2002; Brisdelli et al., 2009; Osmond et al., 2013). Apoptosis is an energy-dependent cascade of molecular events characterized by membrane flip-flop, which leads to the translocation of phosphatidylserine to the outer surface of the membrane, followed by caspase activation and subsequent cleavage of the functional enzyme, PARP, which leads to programmed cell death (Su et al., 2015). Preliminary observations indicate that DETS induces apoptosis as evidenced by staining with AO/EB and Annexin V fluorescence microscopy. Although the mitochondrial and death receptor pathways are distinct, there is considerable crosstalk between them (Kim, 2012). Caspases play an essential role in the apoptotic signal cascade. Caspases 8 and 9 are initiator caspases capable of transducing apoptotic signals by directly activating the downstream executioner caspase 3 (Joe et al., 2002; Chen et al., 2003). The present study clearly demonstrates that DETS induces significant cytotoxicity toward melanoma cells (IC_50_: 24.01 μM) and apoptosis in melanoma cells via the mitochondrial pathway as evidenced by cleavage of caspase 9. As the compound also activates caspase 8, the involvement of death receptor pathway cannot be ruled out, though caspase 8 activation can trigger mitochondrial pathway too. DETS also induced activation of Caspases 8 and 9, clearly demonstrating its mode of action. Though several studies have been reported on the antitumour activity of resveratrol in cancer cells, this is the first study reporting the anticancer activity of DETS, which has the potential to be evaluated as a new anticancer drug.

Different groups have reported that resveratrol blocks cell division by arresting the cells in the S-phase, G1 phase or G2/M phase (Clement et al., 1998; Fuggeta, 2002; Joe et al., 2002; Brisdelli et al., 2009). We have already reported the *in vitro* antioxidant and anticancer activity of another stilbene analog, 3,5-dihydroxy-4-isopropystilbene purified from the cell free culture filtrate of *Bacillus* sp. N strain associated with rhabditid entomopathogenic nematode (Kumar et al., 2013). It has also been reported that resveratrol and a structurally similar molecule, 4-hydroxystilbene induce growth inhibition, apoptosis and S-phase arrest in human melanoma cells (Larrosa et al., 2003). Our results also proved that DETS, a stilbene structurally similar to resveratrol, exhibits subsequent irreversible arrest of melanoma cells in the S-phase, concomitant with a decrease in G0/G1 and G2/M phases, which leads to apoptosis.

Wnt pathway is known to be involved in melanoma progression. Nuclear accumulation of β-catenin, a key regulator of Wnt path way is found to be important in melanoma progression (Widlund et al., 2002; Chien et al., 2009). A recent report from our lab has shown that a fraction, DW-F5 isolated from *Wrightia tinctoria* inhibits the proliferation and progression of melanoma tumor by down-regulating the pivotal molecules such as Brn-2, β-catenin and BRAF along with MITF-M, the master regulator of melanocyte development (Antony et al., 2015). It was very interesting to note that DETS also strongly down-regulate the expression of β-catenin revealing the efficacy of this compound in regulating Wnt signaling. Moreover, β-catenin induced melanoma growth requires the transcriptional activation of its critical downstream target MITF-M. It has also been known that the disruption of this Wnt pathway cause the constitutive over-expression of MITF-M, which establishes the critical role of β-catenin and MITF-M in melanoma cell growth and survival (Larue and Delmas, 2006). An important target gene downstream of Wnt- β-catenin signaling is POU domain transcription factor Brn2, which is found to be over-expressed in melanomas. Studies have shown that BRAF signaling also induces Brn-2 expression, which revealed that Brn2 is a focus for the convergence of two key melanoma associated signaling pathways that are linked to cell proliferation (Goodall et al., 2004). DETS also effectively down-regulated β-catenin, MITF-M, Brn2 and BRAF, which strengthen the hypothesis that this compound is inhibiting the development and proliferation of melanoma cells by regulating WNT-β-catenin pathway, which converge in MITF-M, the master regulator of melanoma signaling and hence, may be the reason why the melanoma cell line A375 exhibited maximum sensitivity toward this compound. The high resistance displayed by SK-MEL-2 against DETS also support our assumption. However, further studies are needed to confirm this hypothesis. An imbalance of ROS on skin is due to factors such as overexposure to sunlight and lack of many essential nutrient intakes, which may eventually lead to skin cancer (Abdul et al., 2014). In our study, DETS recorded substantial antioxidant potential and exhibited significant anticancer activity, especially against melanoma. It was also very interesting to see that the cytotoxic potential of DETS vary depending upon the MITF-M expression status of the melanoma cells.

The present study is the first report on the *in vitro* antioxidant and anticancer properties of DETS. In conclusion, our study indicates that DETS is a strong antioxidant as well as anticancer agent *in vitro*, warranting *in vivo* validation as an anticancer agent, especially against malignant melanoma.

## Author Contributions

LN, SNK, AS: Experiment, AD: Doking experiments, BN, CM: Manuscript writing, RA: Experiment and manuscript writing.

## Conflict of Interest Statement

The authors declare that the research was conducted in the absence of any commercial or financial relationships that could be construed as a potential conflict of interest.

## Footnotes

1 http://www.rcsb.org/pdb/

2 http://pubchem.ncbi.nlm.nih.gov
